# Association of Electronic Nicotine Delivery System Use With Cigarette Smoking Progression or Reduction Among Young Adults

**DOI:** 10.1001/jamanetworkopen.2020.15893

**Published:** 2020-11-24

**Authors:** Jennifer L. Pearson, Eva Sharma, Ning Rui, Michael J. Halenar, Amanda L. Johnson, K. Michael Cummings, Hoda T. Hammad, Annette R. Kaufman, Cindy Tworek, Maciej L. Goniewicz, Heather L. Kimmel, Susanne Tanski, Wilson M. Compton, Hannah Day, Bridget K. Ambrose, Maansi Bansal-Travers, Marushka L. Silveira, David B. Abrams, Jeannie Limpert, Mark J. Travers, Nicolette Borek, Andrew J. Hyland, Cassandra A. Stanton

**Affiliations:** 1 Division of Social and Behavioral Science/Health Administration and Policy, School of Community Health Sciences, University of Nevada, Reno; 2 Department of Health, Behavior and Society, Johns Hopkins Bloomberg School of Public Health, Baltimore, Maryland; 3 Westat, Rockville, Maryland; 4 Office of Science, Center for Tobacco Products Food and Drug Administration, Silver Spring, Maryland; 5 Department of Psychiatry and Behavioral Sciences, Medical University of South Carolina, Charleston; 6 Division of Cancer Control and Population Sciences, National Cancer Institute, National Institutes of Health, Bethesda, Maryland; 7 Division of Cancer Prevention and Population Sciences, Department of Health Behavior, Roswell Park Comprehensive Cancer Center, Buffalo, New York; 8 Division of Epidemiology, Services, and Prevention Research, National Institute on Drug Abuse, National Institutes of Health, Bethesda, Maryland; 9 Norris Cotton Cancer Center and Dartmouth Institute for Health Policy and Clinical Practice, Geisel School of Medicine at Dartmouth, Lebanon, New Hampshire; 10 National Institute on Drug Abuse, National Institutes of Health, Bethesda, Maryland; 11 Kelly Government Solutions, Rockville, Maryland; 12 Department of Social and Behavioral Sciences, NYU College of Global Public Health, New York University, New York; 13 Department of Oncology, Georgetown University Medical Center, Washington, DC

## Abstract

**Importance:**

The prevalence of electronic nicotine delivery systems (ENDS) use, including e-cigarettes, among US young adults (YAs) has raised questions about how these products may affect future tobacco and nicotine use among YAs. Given this prevalence and that young adulthood is a critical period for the establishment of tobacco and nicotine use, it is important to consider the association between ENDS use and cigarette smoking specifically in this age group.

**Objective:**

To examine whether ENDS use frequency or intensity is associated with changes in cigarette smoking among US YA ever smokers during 1 year.

**Design, Setting, and Participants:**

This cohort study used 3 waves of data (2013-2014, 2014-2015, and 2015-2016) from the Population Assessment of Tobacco and Health (PATH) Study, an ongoing longitudinal cohort study of adults and youth. Unweighted 1:6 propensity score matching was used to match participants on wave 1 risk factors for ENDS use at wave 2. The changes in smoking between wave 2 and wave 3 were assessed using the matched sample. In total, 1096 ENDS-naive, ever cigarette–smoking YAs (18-24 years of age) at wave 1 who participated in wave 2 and wave 3 and who had complete data in the PATH Study were included in the analyses, which were conducted from August 2018 to October 2019.

**Exposures:**

Never ENDS use (n = 987), any previous 30-day ENDS use (n = 109), 1 to 5 days of ENDS use in the previous 30 days (n = 75), and 6 or more days ENDS use in the previous 30 days at wave 2 (n = 34).

**Main Outcomes and Measures:**

The analytic sample was selected using multiple variables based on peer-reviewed literature supporting associations with ENDS use. The main outcomes—changes in cigarette smoking behavior between wave 2 and wave 3—were defined using 2 measures: (1) change in smoking frequency, defined as the number of smoking days in the previous 30 days at wave 3 vs wave 2, and (2) change in smoking intensity, defined as the number smoking days in the previous 30 days multiplied by the mean number of cigarettes consumed on smoking days at wave 3 vs wave 2.

**Results:**

The present cohort analyses included 1096 YA ever smokers who were ENDS naive at wave 1. The majority of the sample were women (609 [55.6%]) and White individuals (698 [63.7%]), and the mean (SD) age was 21.4 (1.9) years. In wave 1, 161 YAs (14.7%) were daily smokers in the previous 30 days. After propensity score matching, no statistically significant associations were observed between any definition of wave 2 ENDS use and changes in either the frequency or intensity of smoking at wave 3.

**Conclusions and Relevance:**

In this cohort study of US YA ever smokers, ENDS use was not associated with either decreased or increased cigarette smoking during a 1-year period. However, it is possible that the rapidly evolving marketplace of vaping products may lead to different trajectories of YA cigarette and ENDS use in the future.

## Key Points

**Question:**

Is there an association between electronic nicotine delivery system (ENDS) use and changes in the frequency or intensity of cigarette smoking among ever-smoking young adults?

**Findings:**

In a propensity score matching analysis of 3 years of data from the Population Assessment of Tobacco and Health Study, this cohort study of 1096 young adults found that ENDS use was not associated with an increase or decrease in cigarette smoking frequency or intensity among ever-smoking young adults during 1 year (2014-2015).

**Meaning:**

In this study, new ENDS use among young adult cigarette smokers was not associated with either cigarette smoking increase or decrease during a 1-year period.

## Teaser

This cohort study uses propensity score matching analyses of 3 years of data from the Population Assessment of Tobacco and Health Study to assess whether there is an association between electronic nicotine delivery system use and changes in the frequency or intensity of cigarette smoking among young adults who have ever smoked.

## Introduction

The prevalence of electronic nicotine delivery systems (ENDS) use, including e-cigarettes, among US young adults (YAs) aged 18 to 24 years has raised questions about how these products may affect the future tobacco and nicotine use of YAs.^1,2^ For example, in the first wave (2013-2014) of the US nationally representative Population Assessment of Tobacco and Health (PATH) Study, 32.1% of YAs had ever used ENDS. By the third wave (2015-2016), this proportion had increased to 51.9%.^3^ Data from other US national sources show that young adult never cigarette smokers have a higher prevalence of ever ENDS use compared with adults aged 25 years or older: 9.7% of those aged 18 to 24 years who have never smoked a cigarette have tried an ENDS, compared with 3.2% of never smokers aged 25 years or older.^4^ In addition, results from recent systematic reviews and meta-analyses suggest that ENDS use increases the risk of cigarette smoking initiation in youth and YAs; however, authors have highlighted methods shortcomings that have limited the ability to identify a mechanism (eg, nicotine dependence, propensity to risk taking) driving these associations.^5,6,7,8^ Given those data and that young adulthood is a critical period for the establishment of tobacco and nicotine use,^9,10^ it is important to consider the association between ENDS use and cigarette smoking specifically in this age group.

The National Academies of Science, Engineering, and Medicine (NASEM) recently published a review of ENDS literature concerning their effects on public health,^11^ concluding that ENDS use has the potential for harm reduction among adult cigarette smokers but may actually cause harm if more nonsmokers, especially youth, are induced to initiate smoking who otherwise would not have done so. The authors of the NASEM report found that there is “moderate evidence” to support the assertion that “e-cigarette use increases the frequency and intensity of subsequent tobacco cigarette smoking” among ever-smoking YAs.^11^^(pS-7)^ However, the authors highlight that this conclusion is based on observational studies using limited measures of ENDS use frequency (eg, ever ENDS use and any ENDS use within the previous 30 days) as an exposure. To address this identified gap in evidence, the present study examines the role of ENDS use associated with changes in smoking progression or reduction among YA ever cigarette smokers.

## Methods

### Study Overview

To address the potential of confounding on the association between ENDS use and later cigarette smoking, we used propensity score matching (PSM) to create a sample matched on wave 1 risk factors for ENDS use at wave 2. After PSM, we then examined cigarette smoking increase or decrease between wave 2 and wave 3 using 2 measures: (1) change in smoking frequency, defined as the number of smoking days in the P30D, at wave 3 vs wave 2, and (2) change in smoking intensity, defined as the number of smoking days in the previous 30 days multiplied by the mean number of cigarettes consumed on smoking days, at wave 3 vs wave 2. The wave 3 values were subtracted from the wave 2 values; thus, negative values represent a decrease in cigarette smoking frequency or intensity from wave 2, and positive values represent an increase in cigarette smoking frequency or intensity from wave 2. This study follows the Strengthening the Reporting of Observational Studies in Epidemiology (STROBE) reporting guideline for observational studies.^12^ The study was conducted by Westat and approved by the Westat institutional review board. All participants provided written informed consent, which was obtained in a manner consistent with the Common Rule requirements. Adults who completed the interview received $35 as a thank you for their time.

### Study Design and Population

We used data from the PATH Study Restricted Use File for these analyses. The PATH Study is an ongoing, nationally representative, longitudinal cohort study of adults (aged ≥18 years) and youth (aged 12-17 years) in the US. Data collection occurred in 2013 to 2014 (wave 1), 2014 to 2015 (wave 2), and 2015 to 2016 (wave 3). The study uses audio computer-assisted self-interviews available in English and Spanish to collect self-reported information on tobacco use patterns and associated health behaviors. The study recruitment used a stratified address-based, area-probability sampling design at wave 1 that oversampled adult tobacco users, YAs (aged 18-24 years), and Black adults. An in-person screener was used at wave 1 to randomly select youths and adults from households for participation. At wave 1, the weighted response rate for the household screener was 54.0%. Among screened households, the overall weighted response rate for adults was 74.0% at wave 1, 83.2% at wave 2, and 78.4% at wave 3. The differences in number of completed interviews between wave 1 and subsequent waves reflected attrition due to nonresponse, mortality, and other factors. Further details regarding the PATH Study design and wave 1 methods are published elsewhere.^13^ Details on survey interview procedures, questionnaires, sampling, weighting, and information on accessing the data are discussed in detail elsewhere.^14^ Analyses for the present study were limited to PATH Study participants who were aged 18 to 24 years at wave 1, had exposure data at wave 2, and had data on both outcome variables at wave 3. Participants who aged out of this age range (eg, those who were 24 years old at wave 1 and 25 years old at wave 2) in subsequent data collection waves were retained in these analyses.

### Measures

#### Propensity Matching Variables

Matching variables included age, race/ethnicity, educational level, alcohol use, binge alcohol consumption, marijuana use, other substance use, previous 30-day cigarette smoking (daily, nondaily, and no previous 30-day use), previous 30-day noncigarette combustible use, previous 30-day non-ENDS noncombustible use, previous 12-month quit attempt, intention to quit, tobacco advertising receptivity, e-cigarette harm perceptions, cigarette harm perceptions, nicotine dependence, and the Global Appraisal of Individual Needs–Short Screener substance use, internalizing and externalizing scales. These variables were selected based on published peer-reviewed literature supporting associations with ENDS use. All variables used in the creation of the propensity scores were measured at wave 1 (2013-2014). Citations justifying the inclusion of these variables in the propensity score, variable definitions, and additional information about the construction of the variables are given in eTable 1 in the Supplement. These propensity matching variables were also used as control variables in the treatment estimation models.

#### Exposure Variables

We used previous 30-day ENDS use (yes or no) at wave 2 as the exposure variable. Any young adult ever smoker in our sample who had used an ENDS at least once in the previous 30 days at wave 2 was included in the exposed group. Any young adult ever smoker who remained ENDS naive (never ENDS user) at wave 2 was included in the unexposed group. In a separate analysis, we examined a 3-level previous 30-day ENDS use variable with never previous 30-day ENDS use, 1 to 5 days of use in the previous 30 days, and 6 or more days use in the previous 30 days, to examine the association between increasing levels of ENDS use and the outcomes. These cutoffs (1-5 days, ≥6 days in the previous 30 days) were created based on previous PATH Study publications that identified these cutoffs as meaningful for ENDS use frequency.^15^ Additional information about the construction of the exposure variables is presented in eTable 1 in the Supplement.

#### Outcome Variables

We examined change in cigarette smoking frequency and change in cigarette smoking intensity from wave 2 to wave 3 as our outcome variables. Change in cigarette smoking frequency was calculated by subtracting wave 2 number of days of smoking cigarettes in the previous 30 days from the same value at wave 3. Thus, an increase in the frequency of cigarette smoking at wave 3 is a positive number, a decrease is a negative number, and no change is 0. Similarly, we computed change in cigarette smoking intensity (eg, an estimate of the total number of cigarettes smoked in the previous 30-days) by subtracting cigarette smoking intensity at wave 2 (number of days smoked in the previous 30 days multiplied by the mean number of cigarettes smoked on those days) from the same value at wave 3. Similar to cigarette smoking frequency, a positive value for change in cigarette smoking intensity reflects an increase in intensity from wave 2 to wave 3, and a negative number reflects a decrease in cigarette smoking intensity.

### Statistical Analysis

#### Multiple Imputation

The present analysis focused on 1096 ENDS-naive YAs who had ever smoked at wave 1 who participated in wave 2 and wave 3 of the PATH Study and had ENDS use data at wave 2 and both ENDS and cigarette smoking data at wave 3. Of these YAs, 229 participants were missing at least 1 wave 1 value for variables used in the construction of the propensity scores. We used multiple imputation to infer these missing values so that the full data set from 1096 participants could be used in the PSM. Multiple imputation is a well-established method for inferring the values of missing baseline data.^16^ Participants could be missing wave 1 values used in the construction of the propensity scores because they refused to answer a survey question, for example, or because they did not know the answer to a question; we assumed that these values were missing completely at random. We did not impute values for the exposure or outcome variables. For the sensitivity analysis, we conducted all PSM analyses on a data set in which we did not impute variables and found that the size of the observed effects was similar. We have provided the code used to impute missing data as supplemental material to this publication (eAppendix in the Supplement).

#### Propensity Score Matching

The goal of PSM is to balance the covariate distributions of the exposed (in our case, various levels of P30D ENDS use at wave 2) and unexposed (ENDS naive at wave 2) groups such that the distributions of measured covariates in the 2 groups were similar and confounding on our analyses was minimized. We used the PSM data set to estimate the association between wave 2 ENDS use and wave 3 change in cigarette smoking frequency and intensity.

To obtain the PSM data set, we first estimated the likelihood of previous 30-day ENDS use at wave 1 (the propensity score), then evaluated 1:1 to 1:7 matching approaches with a caliper of 0.2 (ie, the maximum permitted difference in propensity score between matched individuals) to identify the best ratio of individuals in the exposed and unexposed groups.^17,18^ We assessed the quality of the matching approaches using the following criteria: (1) the mean standardized difference of each covariate used in the propensity score postmatching was less than 10% of the pooled prematch and postmatch standard deviation of that variable, and (2) the standardized difference of any covariate did not exceed 25% of the pooled prematch and postmatch standard deviation of the covariate. The standardized difference compares the difference in covariate means between exposed and unexposed groups in units of the pooled standard deviation. Comparing change in standardized difference is a way to assess whether PSM improved balance on covariate distributions in the exposed and unexposed groups. At 1:7 matching, the results failed to meet the above criteria, yielding a larger postmatch standardized difference than 1:6 matching (10.62 vs 8.28) and 3 baseline covariates with a pooled prematch and postmatch standard deviation greater than 25%. Thus, we chose 1:6 matching with replacement and a caliper of 0.2 as our matching approach. eTable 2 in the Supplement shows change in exposed and unexposed standardized difference prematching and postmatching for the 1:6 sample. This resulted in a PSM data set of 105 exposed cases and 483 unexposed cases. In total, 4 exposed cases and 504 unexposed cases were not matched.

#### Regression Analyses

We then used linear regression analyses to estimate different levels of ENDS exposure at wave 2 and the change in cigarette smoking frequency and intensity at wave 3 in the matched sample, using a dummy variable with 1 to 5 days and 6 or more days ENDS use in the previous 30 days to examine the association between escalating amounts of wave 2 ENDS use and the outcomes. We ran 4 different regression models, as follows: (1) never ENDS use vs any previous 30-day ENDS use (n = 588); (2) never ENDS use vs 1 to 5 days ENDS use in the previous 30 days (n = 555); (3) never ENDS use vs 6 or more days ENDS use in the previous 30 days (n = 516); and (4) 1 to 5 days vs 6 or more days ENDS use in the previous 30 days (n = 105). To test whether omission of any of the covariates used in the construction of the propensity score influenced the estimate, 21 regression models were run in which the outcome was measured leaving out 1 covariate at a time. Most estimates with a dropped covariate were within a 10th of the estimate from the full model, but there were 5 covariates used in the construction of the propensity score that caused a dispersion of the final estimates comparing 1 to 5 days ENDS use in the previous 30 days with 6 or more days ENDS use in the previous 30 days by 20% to 80%. Dropping a variable used in the construction of the propensity score did not cause a change in statistical significance; thus, the results suggested that our estimates were reasonably impervious to omission of any of the covariates. The PSM and regression analyses were conducted from August 2018 to October 2019 in R, version 3.3.1 (R Project for Statistical Computing), using the R package MatchIt,^19^ and the code is available in the eAppendix in the Supplement. All tests were 2-tailed, and *P* < .05 was considered statistically significant. All analyses and estimates are unweighted and not representative of the US population.

## Results

### Description of the Sample

Of 1096 YAs in the sample, 609 (55.6%) were young women, 698 (63.7%) were White individuals, and 276 (25.2%) were Hispanic individuals. The mean (SD) age was 21.4 (1.9) years, and 584 (53.3%) had completed at least some college. Of these YAs, 214 (19.5%) were previous 30-day marijuana users and 161 (14.75%) had smoked tobacco daily for the previous 30 days. Within waves, mean smoking frequency and intensity increased with increasing frequency of previous 30-day ENDS at wave 2 (eTable 3 in the Supplement). At wave 3, the mean change in cigarette smoking frequency was close to zero for all groups (−0.4 to 0.7 days). There was greater variation in the mean change in cigarette smoking intensity from wave 2 to wave 3, with a range of −5.0 cigarettes (ENDS naive) to 22.6 or more cigarettes (≥6 days ENDS use in the previous 30 days) (Table 1).

**Table 1.** Sociodemographic, Tobacco, and Other Substance Use Characteristics of the Unweighted Wave 1 Participants by Overall and by Wave 2 ENDS Unexposed and Exposed Groups^a^

Characteristic	Ever cigarette and never ENDS users at wave 1(n = 1096)^b^		Unexposed group: never used ENDS at wave 2 (n = 987)^c^		Exposed group			
					Used ENDS 1-5 d in previous 30 d at wave 2 (n = 75)^d^		Used ENDS ≥6 d in previous 30 d at wave 2 (n = 34)^e^	
	No.	Estimate, %	No.	Estimate, %	No.	Estimate, %	No.	Estimate %^f^
Matching variable (wave 1)								
Mean age	1096	21.4	987	21.5	75	21.1	34	20.8
White	698	63.7	616	62.4	53	70.7	29	85.3
Female	609	55.6	549	55.6	37	49.3	23	67.7
Hispanic	276	25.2	249	25.2	20	26.7	7	20.6
Educational level								
<High school degree	152	13.9	135	13.7	10	13.3	7	20.6
GED	65	5.9	55	5.6	6	8.0	4	11.8
High school degree	295	26.9	259	26.2	24	32.0	12	35.3
Some college or associate degree	435	39.7	396	40.1	28	37.3	11	32.4
≥College	149	13.6	142	14.4	7	9.3	0	0.0
Alcohol use								
Never	197	18.0	173	17.5	16	21.3	8	23.5
Ever, no previous 12 mo	88	8.0	81	8.2	2	2.7	5	14.7
Previous 12 mo, excluding previous 30 d	193	17.6	179	18.1	10	13.3	4	11.8
Previous 30 d	618	56.4	554	56.1	47	62.7	17	50.0
Heavy drinker	148	13.5	122	12.4	21	28.0	5	14.7
Marijuana use								
Never	447	40.8	403	40.8	30	40.0	14	41.2
Ever, no previous 12 mo	295	26.9	271	27.5	15	20.0	9	26.5
Previous 12 mo, excluding previous 30 d	140	12.8	124	12.6	11	14.7	5	14.7
Previous 30 d	214	19.5	189	19.2	19	25.3	6	17.7
Other substance use								
Never	829	75.6	753	76.3	50	66.7	26	76.5
Ever, no previous 12 mo	131	12.0	116	11.8	12	16.0	3	8.8
Previous 12 mo, including previous 30 d	129	11.8	111	11.3	13	17.3	5	14.7
Cigarette smoking								
Daily previous 30-d use	161	14.7	124	12.6	22	29.3	15	44.1
Nondaily previous 30-d use	241	22.0	210	21.3	22	29.3	9	26.5
No previous 30-d use	694	63.3	653	66.2	31	41.3	10	29.4
Previous 30-d non-cigarette combustible use	147	13.4	134	13.6	8	10.7	5	14.7
Previous 30-d non-ENDS noncombustible use	54	4.9	43	4.4	7	9.3	4	11.8
Previous 12-mo quit attempt								
Yes	134	12.2	113	11.5	14	18.7	7	20.6
No	199	18.2	156	15.8	27	36.0	16	47.1
NA	763	69.6	718	72.8	34	45.3	11	32.4
Intention to quit in next 12 mo								
Yes	129	11.8	103	10.4	16	21.3	10	29.4
No	204	18.6	166	16.8	25	33.3	13	38.2
NA	763	69.6	718	72.8	34	45.3	11	32.4
Lifetime GAIN substance use scale								
Low	586	53.5	534	54.1	37	49.3	15	44.1
Moderate	324	29.6	291	29.5	21	28.0	12	35.3
High	186	17.0	162	16.4	17	22.7	7	20.6
Lifetime GAIN externalizing scale								
Low	371	33.9	344	34.9	21	28.0	6	17.7
Moderate	303	27.7	273	27.7	18	24.0	12	35.3
High	422	38.5	370	37.5	36	48.0	16	47.1
Lifetime GAIN internalizing scale								
Low	408	37.2	380	38.5	18	24.0	10	29.4
Moderate	320	29.2	284	28.8	25	33.3	11	32.4
High	368	33.6	323	32.7	32	42.7	13	38.2
Tobacco ad receptivity								
Receptive to cigarette and any tobacco ad other than ENDS	254	23.2	231	23.4	15	20.0	8	23.5
Receptive to ENDS and any tobacco ad other than cigarettes	152	13.9	136	13.8	12	16.0	4	11.8
Receptive to both cigarettes and ENDS and any other products ad	426	38.9	373	37.8	35	46.7	18	52.9
Receptive to other product ad except for cigarettes or ENDS	69	6.3	64	6.5	5	6.7	0	0.0
Not receptive to any tobacco advertising	195	17.8	183	18.5	8	10.7	4	11.8
ENDS perceived harm compared with cigarettes								
Less harmful	463	42.2	405	41.0	42	56.0	16	47.1
About the same	469	42.8	433	43.9	26	34.7	10	29.4
More harmful	68	6.2	61	6.2	3	4.0	4	11.8
Do not know	14	1.3	14	1.4	0	0.0	0	0.0
Have not heard of e-cigarettes	82	7.5	74	7.5	4	5.3	4	11.8
Cigarette perceived harm								
Low	31	2.8	23	2.3	4	5.3	4	11.8
Moderate	117	10.7	100	10.1	10	13.3	7	20.6
High	948	86.5	864	87.5	61	81.3	23	67.7
Nicotine dependence, median	1096	0.0	987	0.0	75	9.4	34	14.1
Outcome variable (wave 3)								
Mean change in number of days smoked cigarettes in previous 30 d, wave 2 to wave 3	1096	0.04	987	0.05	75	−0.4	34	0.7
Mean change in intensity of smoking cigarettes previous 30 d, wave 2 to wave 3	1096	−3.7	987	−5.0	75	1.3	34	22.6

Abbreviations: ENDS, electronic nicotine delivery system; GAIN, Global Appraisal of Individual Needs; GED, General Educational Development.

^a^ Population Assessment of Tobacco and Health Study data from wave 1 to wave 3. Analysis included wave 1 young adults (aged 18-24 years) with data for all 3 waves. Respondent age was calculated based on age at wave 1. Analytic sample included wave 1 ever cigarette smoker and never ENDS users (n = 1585) and had nonmissing data on wave 2 ENDS exposure and wave 3 data on change in number of days smoked between wave 2 and wave 3 (n = 1096). ENDS included e-cigarettes at wave 1, and e-cigarettes, e-cigars, e-pipes, and e-hookah at wave 2 and wave 3. All use definitions refer to any use that includes exclusive or poly use of ENDS. All estimates are unweighted.

^b^ Includes wave 1 ever cigarette users, never ENDS users and had nonmissing data on wave 2 ENDS exposure and wave 3 data on change in number of days smoked between wave 2 and wave 3.

^c^ Includes wave 1 ever cigarette users, wave 2 never ENDS user.

^d^ Includes wave 1 ever cigarette users, wave 2 new ENDS user who used ENDS on 1 to 5 days in the previous 30 days at wave 2.

^e^ Includes wave 1 ever cigarette users, wave 2 new ENDS user who used ENDS on 6 or more days in the previous 30 days at wave 2.

^f^ All estimates in this column have a denominator less than 50 except those with results of 0.0.

The Figure shows how the analytic sample was achieved. We started with 1096 YAs who were ever cigarette smokers but never ENDS users at wave 1. Eligible respondents also had to have complete exposure and outcome data measured at wave 2 and wave 3. Of 1096 YAs, 109 (9.9%) reported at wave 2 ENDS use in the previous 30 days. Of 109 YAs who reported any previous 30-day ENDS use, 75 (68.8%) had used an ENDS 1 to 5 days, and 34 (31.2%) had used an ENDS 6 or more days in the previous 30 days at wave 2.

**Figure.**
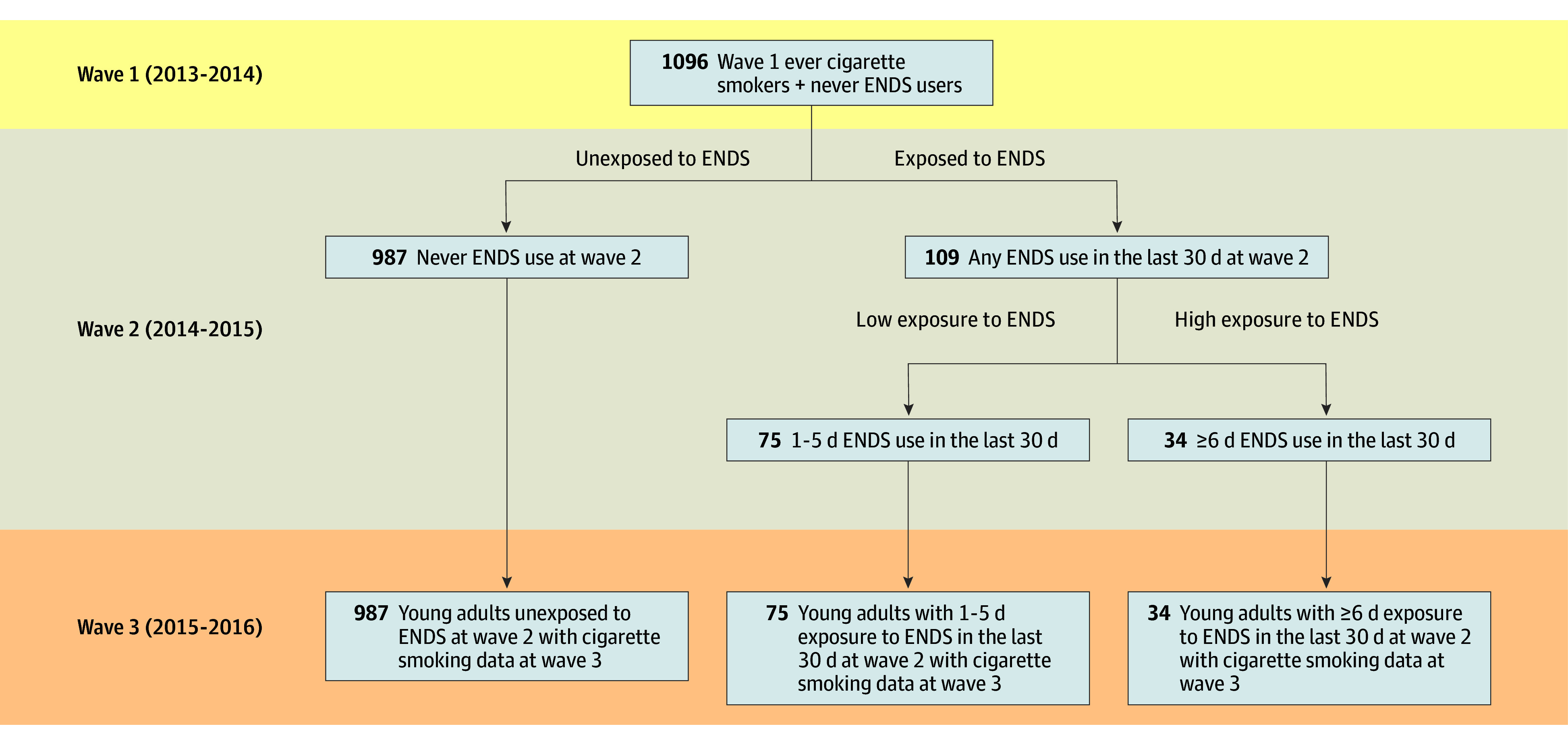
Description of Sample Size and Exposure Status for the Young Adult Analytic Sample From the Population Assessment of Tobacco Health Study Wave 1 to Wave 3. ENDS indicates electronic nicotine delivery system.

There were several notable differences in wave 1 matching variables comparing the ENDS-naive and the previous 30-day ENDS users (Table 1). A greater proportion of individuals who initiated ENDS use at 6 or more days in the previous 30 days were White (85.3% vs 62.4%; *P* = .007), were daily cigarette smokers (44.1% vs 12.6%; *P* < .001), intended to quit using tobacco in the next 12 months (29.4% vs 10.4%; *P* < .001), attempted to quit using any tobacco in the previous 12 months (20.6% vs 11.5%; *P* < .001), were previous 30-day non-ENDS, were noncombusted product users (11.8% vs 4.4%; *P* = .04), and had a higher median tobacco dependence score (14.1% vs 0.0%; *P* < .001) than ENDS-naive participants. More frequent ENDS users were also less educated than the ENDS-naive YAs (eg, 40.1% of ENDS never users had completed at least some college vs 32.4% of individuals who used ENDS for ≥6 days; *P* = .005). A greater proportion of 6 or more days new previous 30-day ENDS users were daily cigarette smokers (42.4% vs 22.8%; *P* = .02), intended to quit using tobacco in the next 12 months (27.3% vs 18.0%; *P* = .03), attempted to quit using any tobacco in the previous 12 months (17.8% vs 18.2; *P* = .008), and had a higher median tobacco dependence score (14.1% vs 0; *P* = .01) compared with ENDS-naive participants.

### Association Between Wave 2 ENDS Use and Wave 3 Changes in Cigarette Smoking

In the PSM data set (Table 2), there were 0.33-day to 1.64-day increases in the number of smoking days in the previous 30 days, depending on the definition of ENDS use at wave 2; none of these changes were associated with ENDS use at wave 2. There were also increases in smoking intensity in the previous 30 days between wave 2 and wave 3, especially among YAs who used ENDS 6 or more days in the previous 30 days compared with never users; however, none of these estimates were associated with ENDS use at wave 2. The estimate for individuals who used ENDS for 6 or more days in the past 30 days, compared with those who never used ENDS, increased by 44.4 cigarettes smoked in the past 30 days, but the increase was not statistically significant.

**Table 2.** Unweighted Association Between Different Levels of ENDS Exposure at Wave 2 and Change in Cigarette Smoking Frequency and Intensity at Wave 3^a^

ENDS exposure at wave 2	Difference in change in cigarette smoking frequency, wave 3 vs wave 2, days	Difference in change in cigarette smoking intensity, wave 3 vs wave 2, cigarettes
Never ENDS use vs any previous 30-d ENDS use (n = 588)	0.71 (−0.94 to 2.37)	17.24 (−13.15 to 47.63)
Never ENDS use vs 1-5 d in previous 30 d (n = 555)	0.33 (−1.47 to 2.14)	6.26 (−28.57 to 41.08)
Never ENDS use vs ≥6 d in previous 30 d (n = 516)	1.41 (−1.35 to 4.17)	44.42 (−8.47 to 97.30)
1-5 d vs ≥6 d in previous 30 d (n = 105)	1.64 (−3.81 to 7.09)	13.67 (−58.94 to 86.27)

Abbreviation: ENDS, electronic nicotine delivery system.

^a^ Population Assessment of Tobacco and Health Study data from waves 1 to 3 among young adult ever cigarette smokers aged 18 to 24 years at wave 1. Wave 3 values were subtracted from wave 2 values so that negative values represent a decrease in cigarette smoking frequency or intensity from wave 2, and positive values represent an increase in cigarette smoking frequency or intensity from wave 2. Results are from each of the 4 regression models defined in the first column.

## Discussion

In this sample of 1096 ENDS-naive ever cigarette smoking US YAs, ENDS use was not associated with either progression or reduction in cigarette smoking during a 1-year period. Previous analyses with 2 waves of PATH Study data used similar PSM models to examine whether the frequency of ENDS use was associated with changes in cigarette smoking frequency among ever and never smoking youths aged 12 to 17 years.^6^ Those models indicated that among wave 1 youth ENDS users, any previous 30-day ENDS use was associated with increased odds of smoking initiation when compared with youths who had not used ENDS. In addition, wave 1 ENDS 1- to 5-day use in the previous 30 days was associated with a decrease in cigarette smoking frequency among ever cigarette smokers, whereas wave 1 6 or more day ENDS use in the previous 30 days was not associated with a change in the frequency of cigarette smoking. The youth study was limited by small samples of ENDS users and a somewhat weak indicator of cigarette smoking (ie, any smoking during the previous 12 months). However, unlike the present YA study, the youth study detected both a significant increase in the risk of transition to ever cigarette smoking and a significant decrease in the number of cigarette smoking days for some definitions of ENDS exposure for a 1-year period. It may be that youth (aged 12-17 years) use ENDS and cigarettes less frequently and more intermittently than YAs and therefore are more sensitive to changes in their early tobacco use patterns. YAs may be more resistant to frequency and intensity changes in their more established tobacco use patterns (including no use); this would be consistent with cross-sectional PATH Study data that show YAs have higher rates of previous 30-day cigarette use compared with youth or adults aged 25 years or older.^3^ We also attempted these analyses with never cigarette smoking YAs at wave 1, but we had an insufficient number of new ENDS users at wave 2 or new cigarette smokers at wave 3 to conduct PSM analyses. The majority of YAs in our sample who had made it to young adulthood as never smokers were unlikely to try ENDS in a subsequent year.

Our null findings could be due to several factors. First, our sample size for these analyses was moderate. A post hoc examination of the minimum detectable effect size given our sample indicated that we had the power to observe a difference of 2.3 days in change in smoking frequency and of 42.2 in change in total cigarettes smoked in a month (intensity) between the exposed and unexposed groups. Although post hoc analyses confirmed that our analyses were adequately powered for all but 1 of the main comparisons, we did not have a large sample of new previous 30-day ENDS users between wave 1 and wave 2, which limited our ability to compare, for example, daily previous 30-day ENDS users with nondaily users. Second, it is possible that different definitions of ENDS use or cigarette smoking could have yielded different outcomes. Third, our null findings could also be due to a true and meaningful null conclusion; using rigorous methods to match wave 1 never ENDS users on baseline ENDS use risk factors, new ENDS use at wave 2 was not associated with cigarette smoking frequency or intensity a year later at wave 3.

### Limitations

This study has limitations. Although matching has gained popularity in substance use research, it is not without potential drawbacks. Previous studies have found that existing PSM methods still have limitations due to their overreliance on observed covariates and possible overestimation of the certainty of findings.^20^ When the number of covariates is limited, substantial hidden bias in the estimate may still exist due to unobserved differences between the treatment and control groups.^21^ In addition, PSM usually requires a large sample size to achieve satisfactory balance in the baseline covariates. We have attempted to address these potential drawbacks by applying PSM to a large longitudinal sample of YAs with a rich assessment of tobacco and other substance use behaviors, including items such as drug and alcohol use, which have been identified as particularly important but frequently overlooked potential confounders in similar studies (eTable 1 in the Supplement),^7^ and reporting prematching and postmatching sample characteristics so that readers may evaluate the efficacy of our matching approach (eTable 2 in the Supplement). However, we acknowledge that hidden bias may yet influence our estimates, especially for the smaller samples of 1 to 5 days and 6 or more days in the previous 30-day e-cigarette users.

Because this is, to our knowledge, the first study of its kind using 3 waves of annual data and a sample of YA ever cigarette smokers, it is important that future researchers replicate these analyses in other data sets and over time in the PATH Study as well as examine the associations between different definitions of ENDS use and subsequent cigarette smoking. Because the wave 2 ENDS use data were collected in 2015, these data may not be reflective of rapidly changing dynamics in vaping device technology and market sales. New data on ENDS sales show a marked increase in unit sales in 2016 with the introduction of MarkTen, and a subsequent dramatic change in 2017 with the introduction of the pod-based JUUL and similar products (ie, Suorin) to the market.^22,23^ Specifically, JUUL dominated the 2018 brick-and-mortar ENDS marketplace, delivering nicotine on par with a cigarette,^24,25^ which suggests a potential for higher abuse liability as well as potential greater utility as a quit method for cigarette smokers. Analyses of data after 2018 may find different associations between ENDS use and smoking than those reported here.

## Conclusions

In this study, new ENDS use among YA cigarette smokers was not associated with either cigarette smoking increase or decrease for 1 year, from 2014 to 2015. However, it is possible that the rapidly evolving marketplace of vaping products may lead to different trajectories of YA cigarette and ENDS use in the future.

## Notes

Supplement. **eTable 1.** Matching, Exposure, and Outcome Variables; Justification for Inclusion of Matching Variables; and Variable Definition and Construction **eTable 2.** A Comparison of Means, Standard Deviations, and Absolute Standard Differences by W2 ENDS Exposure Status Prematch and Postmatch Sample **eTable 3.** Unweighted Mean Number of Days Smoking in P30D and Mean Number of Cigarettes Smoked in P30D at Waves 2 and 3 Among Wave 1, 18-24 Yeal Old Young Adult Participants in the Population Assessment of Tobacco and Health Study (N=1096) **eAppendix.** Multiple Imputation Code for SAS Statistical Software
eReferences.
