# Supplementary material for: Association of Electronic Nicotine Delivery System Use With Cigarette Smoking Progression or Reduction Among Young Adults
Source: JAMA Netw Open. 2020 Nov 24;3(11):e2015893. doi: 10.1001/jamanetworkopen.2020.15893 (PMC7686868; doi:10.1001/jamanetworkopen.2020.15893)
Supplement: Supplement. — eTable 1. Matching, Exposure, and Outcome Variables; Justification for Inclusion of Matching Variables; and Variable Definition and Construction eTable 2. A Comparison of Means, Standard Deviations, and Absolute Standard Differences by W2 ENDS Exposure Status Prematch and Postmatch Sample eTable 3. Unweighted Mean Number of Days Smoking in P30D and Mean Number of Cigarettes Smoked in P30D at Waves 2 and 3 Among Wave 1, 18-24 Yeal Old Young Adult Participants in the Population Assessment of Tobacco and Health Study (N=1096) eAppendix. Multiple Imputation Code for SAS Statistical Software eReferences. [file jamanetwopen-e2015893-s001.pdf]

Supplementary Online Content

Pearson JL, Sharma E, Rui N, et al. Association of electronic nicotine delivery system use with cigarette smoking progression or reduction among young adults. *JAMA Netw Open*. 2020;3(11):e2015893. doi:10.1001/jamanetworkopen.2020.15893

eTable 1. Matching, Exposure, and Outcome Variables; Justification for Inclusion of Matching Variables; and Variable Definition and Construction

eTable 2. A Comparison of Means, Standard Deviations, and Absolute Standard Differences by W2 ENDS Exposure Status Prematch and Postmatch Sample

eTable 3. Unweighted Mean Number of Days Smoking in P30D and Mean Number of Cigarettes Smoked in P30D at Waves 2 and 3 Among Wave 1, 18-24 Year Old Young Adult Participants in the Population Assessment of Tobacco and Health Study (N=1096)

eAppendix. Multiple Imputation Code for SAS Statistical Software

eReferences.

This supplementary material has been provided by the authors to give readers additional information about their work.

© 2020 Pearson JL et al. *JAMA Network Open*.

eTable 1. Matching, Exposure, and Outcome Variables; Justification for Inclusion of Matching Variables; and Variable Definition and Construction

Variable	Variable definition	Variable/instrument note
Wave 1 matching variable		
Sex[1,2-4]	Female (yes or no)	NA
Age[1,2,4,5]	Continuous; aged 18-24 y	NA
Race/ethnicity[4,6,7]	Identifies as White, non-White or multirace; Hispanic ethnicity (yes or no)	NA
Educational level[1,8,9]	<High school degree, GED, high school degree, some college or associate degree, ≥college	NA
Alcohol use[10]	Defined as never, ever (excluding P12M), P12M (excluding P30D), and P30D use	NA
Heavy drinking[4,7,11]	Heavy drinking (yes or no) defined as 4 or more drinks on drinking days for females and 5 or more drinks on drinking days for males, whereas nondrinkers were coded as "not asked"	NA
Marijuana use[4,10]	Defined as never, ever (excluding P12M), P12M (excluding P30D), and P30D use	NA
Other drug use[7]	Defined as never, ever (excluding P12M), P12M (excluding P30D), and P30D use of cocaine or crack, stimulants, heroin, inhalants, solvents, hallucinogens, or nonprescription use of prescription drugs such as methylphenidate, amphetamine and dextroamphetamine (Adderall), painkillers, sedatives, or tranquilizers	NA
Cigarette use[1,3]	Defined as no use in the P30D, nondaily P30D use, and daily P30D use	NA
Noncigarette combusted use[4,12]	Defined as P30D use of traditional cigars, cigarillos, filtered cigars, hookah, or pipe except cigarettes	NA
Non-ENDS noncombusted use[4,12]	Defined as P30D use of smokeless tobacco, snus, or dissolvable tobacco except e-cigarettes	NA
GAIN-SS substance use scale[13]	Assessed lifetime count of substance use disorder symptoms (eg, When was the last time you…used substances at least weekly, spent a lot of time obtaining substances, reduced involvement in activities due to the use of substances, had problems with withdrawal [7 items])	The following severity threshold cut points were used, counting positive responses to any item during lifetime as a "symptom" within the set of items screening for internalizing, externalizing, and substance use problems: 0-1 symptoms (no/low), 2-3 symptoms (moderate), and ≥4 symptoms (high)

© 2020 Pearson JL et al. *JAMA Network Open*.

Variable	Variable definition	Variable/instrument note
GAIN-SS internalizing or externalizing problems scales[14]	Assessed internalizing problems (eg, "When was the last time that you had significant problems with…Feeling very trapped, lonely, sad, blue, depressed, or hopeless about the future?" [4 items])<br>Assessed externalizing problems (eg, "When was the last time that you did the following things 2 or more times…Lied or conned to get things you wanted or to avoid having to do something? Were a bully or threatened other people?" [7 items])	The following severity threshold cut points were used, counting positive responses to any item during lifetime as a "symptom" within the set of items screening for internalizing, externalizing, and substance use problems: 0-1 symptoms (no/low), 2-3 symptoms (moderate), and 4 symptoms (high).
Previous 12-mo tobacco quit attempt[1]	Quit attempt of any tobacco product in the P12M (yes/no/"not asked" for respondents who were skipped out)	NA
Intention to quit tobacco[1,15]	Intention to quit using any tobacco products within 12 mo	NA
Tobacco advertising receptivity[6,16]	Tobacco advertising receptivity measured on 5 levels: 1 = receptive to advertising for cigarettes and any other products except for e-cigarettes; 2 = receptive to advertising for e-cigarettes and any other products except for cigarettes; 3 = receptive to advertising for both cigarettes and e-cigarettes and any other products; 4 = receptive to advertising for other products except for cigarettes or e-cigarettes; 5 = not receptive to advertising for any tobacco product	Respondents were shown a stratified random set of 20 tobacco advertisements (5 for each of the following products: cigarettes, e-cigarettes, cigars, and smokeless products) from 959 recently used print, direct mail, Internet, and television advertisements (ad). Respondents reported whether they had (1) seen the ad in the previous 12 mo or (2) liked the ad, regardless of prior exposure to the ad. Receptivity was product class–specific (eg, receptive to ENDS ad or cigarette ad) and defined as P12M recall or liking of each type of ad. More information available elsewhere.[17]
Cigarette harm perception[18,19]	Perceived harm from one's own cigarette use (low, medium, or high harm)	NA
ENDS harm perceptions[2,4,6,20]	Perception of e-cigarette harm relative to cigarettes (less, about the same, more harmful, don't know, haven't heard of the product)	NA
Nicotine dependence[21]	Composite tobacco dependence summary measure, reduced from several multi-item measures of tobacco dependence using item response theory analyses, and representing a common 16-item tobacco dependence measure from tobacco product such as	Each of the original 16 items were rescaled to 3-level response categories (0, 50, 100) and summed to create a continuous variable with a range of 0-1600 and representing, respectively, low to high levels

© 2020 Pearson JL et al. *JAMA Network Open*.

Variable	Variable definition	Variable/instrument note
	cigarettes, e-cigarettes, cigars, pipe, hookah, and smokeless tobacco	of dependence. Nonusers were coded as 0.
Wave 2 exposure		
ENDS exposure	Never ENDS use, 1-5 d use, and ≥6 d ENDS use in the P30D. An ENDS product was defined as an e-cigarette, e-cigar, e-pipe, e-hookah, personal vaporizer, vape pen, or hookah pen	NA
Wave 3 outcome		
Change in cigarette smoking frequency	Change in the number of days smoked cigarettes in P30D, W3 vs W2	All P30D nonsmokers were assigned a value of 0 (smoked 0 d in P30D)
Change in cigarette smoking intensity	Change in cigarette smoking intensity (frequency multiplied by quantity) in the P30D, W3 vs W2	Smoking intensity at each wave was defined as number of days smoked a cigarette multiplied by the mean number of cigarettes smoked per day in P30D

Abbreviations: ENDS, electronic nicotine delivery system; GAIN-SS, Global Appraisal of Individual Needs–Short Screener; GED, General Educational Development; NA, not applicable; P12M, previous 12 months; P30D, previous 30 days; W2, wave 2.

© 2020 Pearson JL et al. *JAMA Network Open*.

eTable 2. A Comparison of Means, Standard Deviations, and Absolute Standard Differences by W2 ENDS Exposure Status Prematch and Postmatch Sample

	P30D ENDS use at W2 (n=109)		No ENDS use at W2 (n=987)		
**Variable**	**Mean**	**SD**	**Mean**	**SD**	**Absolute Standardized Difference***
**Pre-Match Sample**					
Age	21.01	1.90	21.49	1.90	25.18
Race	0.25	0.43	0.38	0.48	27.87
Sex	0.55	0.50	0.56	0.50	1.16
Ethnicity	0.25	0.43	0.25	0.43	1.05
Education	2.08	1.16	2.36	1.20	23.47
Alcohol use	2.08	1.24	2.13	1.15	3.85
Binge drinking	0.76	0.43	0.88	0.33	30.09
Cannabis use	1.20	1.20	1.10	1.14	8.69
Other substance use	0.55	0.96	0.39	0.81	18.01
Cigarette smoking	2.04	0.85	2.54	0.71	63.89
P30D non-cigarette combustible use	0.88	0.33	0.86	0.34	4.94
P30D non-ENDS non-combustible use	0.90	0.30	0.96	0.20	22.22
Past 12-month quit attempt	1.22	0.75	1.61	0.68	54.76
Intention to quit	1.17	0.79	1.62	0.67	61.34
GAIN substance use scale	0.74	0.80	0.62	0.75	15.49
GAIN internalizing scale	1.16	0.81	0.94	0.84	25.91
GAIN externalizing scale	1.23	0.82	1.03	0.85	24.25
Tobacco advertising receptivity	2.70	1.18	2.83	1.36	10.39
E-cigarette harm perceptions	0.75	1.10	0.90	1.09	13.91
Cigarette harm perceptions	1.70	0.60	1.85	0.42	29.96
Nicotine dependence	25.04	29.43	11.28	21.63	53.26
**Average standardized difference**					**24.75**

© 2020 Pearson JL et al. *JAMA Network Open*.

Post-Match Sample					
	**P30D ENDS use at W2 (n=105)**		**No ENDS use at W2 (n=483)**		
**Variable**	**Mean**	**SD**	**Mean**	**SD**	**Absolute Standardized Difference**
Age	21.03	1.91	21.11	1.89	10.04
Race	0.26	0.44	0.30	0.46	0.33
Sex	0.54	0.50	0.54	0.50	0.67
Ethnicity	0.25	0.43	0.25	0.43	10.34
Education	2.10	1.15	2.22	1.21	5.53
Alcohol use	2.06	1.25	2.12	1.17	6.02
Binge drinking	0.78	0.42	0.81	0.40	2.29
Cannabis use	1.21	1.20	1.18	1.19	3.29
Other substance use	0.53	0.94	0.50	0.90	24.38
Cigarette smoking	2.08	0.84	2.28	0.81	6.18
Current non-cigarette combustible use	0.88	0.33	0.86	0.35	5.86
Current non-ENDS non-combustible use	0.90	0.29	0.92	0.27	19.50
Past 12-month quit attempt	1.25	0.74	1.40	0.77	23.64
Intention to quit	1.21	0.78	1.39	0.77	8.44
GAIN substance use scale	0.75	0.81	0.69	0.78	2.98
GAIN internalizing scale	1.12	0.80	1.10	0.84	1.15
GAIN externalizing scale	1.20	0.83	1.19	0.83	2.73
Tobacco advertising receptivity	2.72	1.18	2.76	1.30	2.45
ENDS harm perceptions	0.77	1.11	0.80	0.98	15.53
Cigarette harm perceptions	1.70	0.59	1.79	0.49	18.42
Nicotine dependence	23.01	27.94	18.04	26.08	10.04
**Average standardized difference**					**8.28**

Notes – SD = standard deviation; P30D = past 30-day

*standardized difference = $\frac{100(\bar{x}_{treated} - \bar{x}_{control})}{\sqrt{\frac{s^2_{treated} + s^2_{control}}{2}}}$

© 2020 Pearson JL et al. *JAMA Network Open*.

eTable 3. Unweighted Mean Number of Days Smoking in P30D and Mean Number of Cigarettes Smoked in P30D at Waves 2 and 3 Among Wave 1, 18-24 Year Old Young Adult Participants in the Population Assessment of Tobacco and Health Study (N=1096)

	Wave 2: Smoking days in the P30D		Wave 3: Smoking days in the P30D		Wave 2: Number of cigarettes smoked in P30D		Wave 3: Number of cigarettes smoked in P30D	
	Mean	SD	Mean	SD	Mean	SD	Mean	SD
Never ENDS use (N=987)	4.89	10.14	4.94	10.29	51.99	174.15	46.96	146.86
1-5 days in P30D (N=75)	12.71	13.75	12.33	13.44	123.01	191.26	124.28	192.44
6+ days in P30D (N=34)	16.68†	12.96†	17.41†	12.99†	138.38†	172.42†	161.03†	195.04†

Notes: Analysis included Wave 1 Young Adults (18-24 year olds) with data at all three waves. Respondent age was calculated based on age at Wave 1.
Analytic sample included Wave 1 ever cigarette smoker and never ENDS users (1,585) and had non-missing data on Wave 2 ENDS exposure and Wave 3 data on change in number of days smoked between Wave 2 and Wave 3 (1,096).
ENDS includes e-cigarettes at W1, and e-cigarettes, e-cigars, e-pipes, and e-hookah at W2 and W3.
P30D = past 30 days

All use definitions refer to any use that includes exclusive or poly use of ENDS.
All estimates are unweighted.

† Estimates with denominator less than 50 are flagged.

© 2020 Pearson JL et al. *JAMA Network Open*.

eAppendix. Multiple Imputation Code for SAS Statistical Software

```
*output a table of the missing data patterns present in the data file, which shows 229 cases have missing
data on at least one w1 covariate;
proc mi data=ECIG.ya2 nimpute=0 ;
var R01R_A_AGE R01R_A_RACECAT2A R01R_A_SEXA R01R_A_ETHCAT2A R01R_A_EDUC_CAT5A R01R_A_USESTATUS_ALCA
R01R_A_PAST30_ALCBINGEA R01R_A_USESTATUS_MJA R01R_A_USESTATUS_OTHDRUGA R01R_A_USESTATUS_CIGSB
R01R_A_P30D_COMBUST_NOCIGA
R01R_A_P30D_NONCOMBUST_NOECIGA R01R_A_QUIT_1PLUS_PY_CAT3A R01R_A_PLANQUIT_12M_CAT3A
R01R_A_GAINSUSCREEN_LIFEA R01R_A_GAININTSCREEN_LIFEA R01R_A_GAINEXTSCREEN_LIFEA R01R_A_RECEPT_CAT5A
R01R_A_ECIGHARMVCIGA R01R_A_CIGHARM_CAT3A R01R_A_DPNDSCL_INCLNA;
ods select misspattern;
run;

*Use PROC MI to specify the imputation model to be used and the number of imputed datasets =5;
PROC MI DATA=ECIG.ya2 OUT=IMPUTED NIMPUTE=5 SEED= 95243189
      ROUND= . . . . . . . . 1 . .;
      MCMC INITIAL=EM(MAXITER=500) NBITER=500 NITER=200;
var R01R_A_AGE
R01R_A_RACECAT2A
R01R_A_SEXA
R01R_A_ETHCAT2A
R01R_A_EDUC_CAT5A
R01R_A_USESTATUS_ALCA
R01R_A_PAST30_ALCBINGEA
R01R_A_USESTATUS_MJA
R01R_A_USESTATUS_OTHDRUGA
R01R_A_USESTATUS_CIGSB
R01R_A_P30D_COMBUST_NOCIGA
R01R_A_P30D_NONCOMBUST_NOECIGA
R01R_A_QUIT_1PLUS_PY_CAT3A
R01R_A_PLANQUIT_12M_CAT3A
R01R_A_GAINSUSCREEN_LIFEA
R01R_A_GAININTSCREEN_LIFEA
R01R_A_GAINEXTSCREEN_LIFEA
R01R_A_RECEPT_CAT5A
R01R_A_ECIGHARMVCIGA
R01R_A_CIGHARM_CAT3A
R01R_A_DPNDSCL_INCLNA;
RUN;
```

© 2020 Pearson JL et al. *JAMA Network Open*.

```
*Compute the average of imputed values from 5 models for each covariate;
proc means data=IMPUTED mean noprint nway;
output out=temp mean=;
var R01R_A_AGE
R01R_A_RACECAT2A
R01R_A_SEXA
R01R_A_ETHCAT2A
R01R_A_EDUC_CAT5A
R01R_A_USESTATUS_ALCA
R01R_A_PAST30_ALCBINGEA
R01R_A_USESTATUS_MJA
R01R_A_USESTATUS_OTHDRUGA
R01R_A_USESTATUS_CIGSB
R01R_A_P30D_COMBUST_NOCIGA
R01R_A_P30D_NONCOMBUST_NOECIGA
R01R_A_QUIT_1PLUS_PY_CAT3A
R01R_A_PLANQUIT_12M_CAT3A
R01R_A_GAINSUSCREEN_LIFEA
R01R_A_GAININTSCREEN_LIFEA
R01R_A_GAINEXTSCREEN_LIFEA
R01R_A_RECEPT_CAT5A
R01R_A_ECIGHARMVCIGA
R01R_A_CIGHARM_CAT3A
R01R_A_DPNDSCL_INCLNA;
class PID;
run;

*Compute the distriution of average imputed values for all covariates;
proc means data=temp maxdec=2;
var R01R_A_AGE
R01R_A_RACECAT2A
R01R_A_SEXA
R01R_A_ETHCAT2A
R01R_A_EDUC_CAT5A
R01R_A_USESTATUS_ALCA
R01R_A_PAST30_ALCBINGEA
R01R_A_USESTATUS_MJA
R01R_A_USESTATUS_OTHDRUGA
R01R_A_USESTATUS_CIGSB
R01R_A_P30D_COMBUST_NOCIGA
R01R_A_P30D_NONCOMBUST_NOECIGA
```

© 2020 Pearson JL et al. *JAMA Network Open*.

```
R01R_A_QUIT_1PLUS_PY_CAT3A
R01R_A_PLANQUIT_12M_CAT3A
R01R_A_GAINSUSCREEN_LIFEA
R01R_A_GAININTSCREEN_LIFEA
R01R_A_GAINEXTSCREEN_LIFEA
R01R_A_RECEPT_CAT5A
R01R_A_ECIGHARMVCIGA
R01R_A_CIGHARM_CAT3A
R01R_A_DPNDSCL_INCLNA;
run;

*Round the imputed values to proper integers for discrete variables;
data imputed1;
set temp;
drop _TYPE_ _FREQ_;
      array a[*] R01R_A_RACECAT2A
R01R_A_SEXA
R01R_A_ETHCAT2A
R01R_A_EDUC_CAT5A
R01R_A_USESTATUS_ALCA
R01R_A_PAST30_ALCBINGEA
R01R_A_USESTATUS_MJA
R01R_A_USESTATUS_OTHDRUGA
R01R_A_USESTATUS_CIGSB
R01R_A_P30D_COMBUST_NOCIGA
R01R_A_P30D_NONCOMBUST_NOECIGA
R01R_A_QUIT_1PLUS_PY_CAT3A
R01R_A_PLANQUIT_12M_CAT3A
R01R_A_GAINSUSCREEN_LIFEA
R01R_A_GAININTSCREEN_LIFEA
R01R_A_GAINEXTSCREEN_LIFEA
R01R_A_RECEPT_CAT5A
R01R_A_ECIGHARMVCIGA
R01R_A_CIGHARM_CAT3A;
      do i = 1 to dim(a);
            if a[i] <0.5 then a[i] = 0;
            else if 0.5<=a[i]<1.5 then a[i] = 1;
            else if 1.5<=a[i]<2.5 then a[i] = 2;
            else if 2.5<=a[i]<3.5 then a[i] = 3;
            else if 3.5<=a[i]<4.5 then a[i] = 4;
            else if a[i]>=4.5 then a[i] = 5;
```

© 2020 Pearson JL et al. *JAMA Network Open*.

```
        end;
if R01R_A_DPNDSCL_INCLNA<0 then R01R_A_DPNDSCL_INCLNA=0;
drop i;
run;
```

© 2020 Pearson JL et al. *JAMA Network Open*.

eReferences

1. Ramo DE, Young-Wolff KC, Prochaska JJ. Prevalence and correlates of electronic-cigarette use in young adults: Findings from three studies over five years. *Addictive behaviors.* 2015;41:142-147.
2. Choi K, Forster J. Characteristics associated with awareness, perceptions, and use of electronic nicotine delivery systems among young us midwestern adults. *American journal of public health.* 2013;103(3):556-561.
3. Hampson SE, Andrews JA, Severson HH, Barckley M. Prospective predictors of novel tobacco and nicotine product use in emerging adulthood. *Journal of Adolescent Health.* 2015;57(2):186-191.
4. Saddleson ML, Kozlowski LT, Giovino GA, et al. Risky behaviors, e-cigarette use and susceptibility of use among college students. *Drug and alcohol dependence.* 2015;149:25-30.
5. Pokhrel P, Little MA, Fagan P, Muranaka N, Herzog TA. Electronic cigarette use outcome expectancies among college students. *Addictive behaviors.* 2014;39(6):1062-1065.
6. Pokhrel P, Fagan P, Kehl L, Herzog TA. Receptivity to e-cigarette marketing, harm perceptions, and e-cigarette use. *American journal of health behavior.* 2015;39(1):121-131.
7. Sutfin EL, McCoy TP, Morrell HE, Hoeppner BB, Wolfson M. Electronic cigarette use by college students. *Drug and alcohol dependence.* 2013;131(3):214-221.
8. Coleman BN, Apelberg BJ, Ambrose BK, et al. Association between electronic cigarette use and openness to cigarette smoking among us young adults. *Nicotine Tobacco Res.* 2014;17(2):212-218.
9. Czoli CD, Hammond D, White CM. Electronic cigarettes in canada: Prevalence of use and perceptions among youth and young adults. *Can J Public Health.* 2014;105(2):e97-e102.
10. Cohn A, Villanti A, Richardson A, et al. The association between alcohol, marijuana use, and new and emerging tobacco products in a young adult population. *Addictive behaviors.* 2015;48:79-88.
11. Bartoli F, Carretta D, Crocamo C, et al. Prevalence and correlates of binge drinking among young adults using alcohol: A cross-sectional survey. *BioMed research international.* 2014;2014.
12. Sutfin EL, Reboussin BA, Debinski B, Wagoner KG, Spangler J, Wolfson M. The impact of trying electronic cigarettes on cigarette smoking by college students: A prospective analysis. *American journal of public health.* 2015;105(8):e83-e89.
13. Richter L, Pugh BS, Smith PH, Ball SA. The co-occurrence of nicotine and other substance use and addiction among youth and adults in the United States: Implications for research, practice, and policy. *The American journal of drug and alcohol abuse.* 2017;43(2):132-145.
14. Cummins SE, Zhu S-H, Tedeschi GJ, Gamst AC, Myers MG. Use of e-cigarettes by individuals with mental health conditions. *Tob Control.* 2014:tobaccocontrol-2013-051511.
15. Rutten LJF, Blake KD, Agunwamba AA, et al. Use of e-cigarettes among current smokers: Associations among reasons for use, quit intentions, and current tobacco use. *Nicotine Tobacco Res.* 2015;17(10):1228-1234.
16. Thrul J, Lisha NE, Ling PM. Tobacco marketing receptivity and other tobacco product use among young adult bar patrons. *J Adolesc Health.* 2016;59(6):642-647.
17. Pierce JP, Sargent JD, White MM, et al. Receptivity to tobacco advertising and susceptibility to tobacco products. *Pediatrics.* 2017;139(6):e20163353. 10.1542/peds.2016-3353 PMC5470502 conflicts of interest to disclose.
18. Hummel K, Hoving C, Nagelhout GE, et al. Prevalence and reasons for use of electronic cigarettes among smokers: Findings from the international tobacco control (itc) netherlands survey. *International Journal of Drug Policy.* 2015;26(6):601-608.
19. Kong G, Morean ME, Cavallo DA, Camenga DR, Krishnan-Sarin S. Reasons for electronic cigarette experimentation and discontinuation among adolescents and young adults. *Nicotine Tobacco Res.* 2014;17(7):847-854.
20. Choi K, Forster JL. Beliefs and experimentation with electronic cigarettes: A prospective analysis among young adults. *American journal of preventive medicine.* 2014;46(2):175-178.
21. Strong DR, Pearson J, Ehlke S, et al. Indicators of dependence for different types of tobacco product users: Descriptive findings from Wave 1 (2013-2014) of the population assessment of tobacco and health (path) study. *Drug Alcohol Depend.* 2017;178:257-266. 10.1016/j.drugalcdep.2017.05.010

© 2020 Pearson JL et al. *JAMA Network Open*.
